# mSphere of Influence: When a sequencer is more than a sequencer

**DOI:** 10.1128/msphere.00433-24

**Published:** 2024-09-10

**Authors:** R. Blake Billmyre

**Affiliations:** 1 Department of Pharmaceutical and Biomedical Sciences, College of Pharmacy, University of Georgia, Athens, Georgia, USA; 2 Department of Infectious Diseases, College of Veterinary Medicine, University of Georgia, Athens, Georgia, USA; 3 Department of Microbiology, Franklin College of Arts and Sciences, University of Georgia, Athens, Georgia, USA; 4 Department of Genetics, Franklin College of Arts and Sciences, University of Georgia, Athens, Georgia, USA; University of Georgia, Athens, Georgia, USA

**Keywords:** TN-seq, genomics, yeast, DNA sequencing

## Abstract

Blake Billmyre uses functional genomics to help understand the biology of fungal pathogens, with an emphasis on evolution of virulence relevant traits and drug resistance. In this mSphere of Influence article, he reflects on how two papers (Liachko et al., “High-resolution mapping, characterization, and optimization of autonomously replicating sequences in yeast,” Genome Research, 2013, and Guo et al., “Integration profiling of gene function with dense maps of transposon integration,” Genetics, 2013) impacted his research trajectory and goals. These articles show the power of creative use of sequencing as a tool to drive understanding of fundamental biology.

## COMMENTARY

Chance plays an enormous role in all of our scientific trajectories. One of the many unexpected changes in my path came from a chance encounter at a meeting. As a graduate student, I attended an EMBO Comparative Genomics of Eukaryotic Microbes meeting. The meeting was in Spain and a several-hour drive away from the airport in Barcelona. I had made the amateur mistake of checking my bag for the flight, which predictably meant that my bag was still in the United States when I landed. Around the time my bag finally arrived more than 24 hours later, Maitreya Dunham gave a talk about her lab’s work using deep mutational scanning to define the nucleotide sequence basis of autonomously replicating sequences (ARSs) in the budding yeast *Saccharomyces cerevisiae* (1). ARSs are specific sequences that act as DNA replication origins. The Dunham group had taken a clever approach to screen for sequences capable of acting as ARSs by cloning pools of random genomic DNA fragments into a plasmid lacking an ARS, transforming it into *S. cerevisiae*, growing the pools of transformants in competition, and finally sequencing the plasmid inserts as a direct read out of ARS fitness. They went on to identify a short 100-bp core sequence and construct a new library with saturated mutations of this original short core sequence, retesting fitness of all possible single base mutations within that 100-bp sequence.

Perhaps it was in part the newly arrived clean clothes, but this talk was a paradigm shift for me. I worked in genomics, but I primarily looked at natural sequence variation and used comparative genomics approaches. I had never heard of deep mutational scanning. Rather than purely looking at a genome sequence, the Dunham group was using high-throughput sequencing to measure the frequency of different individuals within a pool as a direct experimental output, something I had never even considered before. I returned from the meeting determined to find my own version of the ARS screen, using my skill set in sequencing and informatics to understand biology in a cool new way. At the time, students in the neighboring Alspaugh lab at Duke were using *Agrobacterium* to generate large numbers of random insertional mutants in *Cryptococcus neoformans*. They would then screen their mutants for phenotypes of interest and map the hits using whole-genome sequencing (2, 3). It occurred to me that the vast majority of the sequencing depth in this type of experiment was unwanted genomic sequence, when they really just wanted to know the location of their inserts. Alternately, using specific primers to amplify just the inserts prior to sequencing could allow very high-throughput determination of insert location or, with a little extension, even insert frequency and fitness similar to the Dunham lab’s example.

I excitedly brought this idea to a senior colleague who immediately asked me if I was describing TN-seq, a well-known (by others) functional genomic technique. TN-seq uses mobile DNA transposons to generate random insertional mutations throughout a genome, ideally with a single insertional mutant per cell. The transposon insertions can then be amplified and sequenced using high-throughput sequencing, achieving exactly what I had wanted to do, but in a better and more tractable way. While the technique originated in bacteria (4–7), my senior colleague pointed me to a paper by Henry Levin’s group, which brought TN-seq to fungi (8). Henry’s group used a modified Hermes transposon that originated in house flies and mobilized it throughout the fission yeast *Schizosaccharomyces pombe* genome. Using dense insertion maps, they were able to predict essential genes across the genome and also uncover factors that affected transposon insertions, including sequence bias and nucleosome occupancy. Both outputs are only possible because of the very high scale enabled by TN-seq relative to earlier mapping methods. The Levin lab later used this approach to examine genes involved in heterochromatin formation (9) and general fitness (10).

The Levin lab’s paper went on to inspire many other fungal geneticists to build their own TN-seq systems in their own fungus, and we now have functional TN-seq systems in the model budding yeast *S. cerevisiae* (11), and the pathogenic fungi *Candida albicans* (12), and *Nakaseomyces glabratus* (13). This system is an enormous boon particularly for those working on non-model organisms where mutant collections tend to be rare. I can count myself among those inspired: I joined SaraH Zanders for my postdoc to use the *S. pombe* TN-seq system developed by the Levin lab to study genes required for sexual reproduction (14) and develop a TN-seq system for the human fungal pathogen *Cryptococcus neoformans* (15), my original goal after I returned from seeing Maitreya’s talk in Spain. My own lab continues to use TN-seq in *C. neoformans*, with an eye toward understanding species-level differences across the *Cryptococcus* genus. Along with the new generation of clustered regularly interspaced short palindromic repeat-guided (CRISPR) tools, TN-seq promises to enable powerful genetic screens in systems where they would otherwise be impractical.
